# Impact of Salpingotomy on Intrauterine Pregnancy and Recurrent Ectopic Pregnancy Rates: A Meta-Analysis

**DOI:** 10.7759/cureus.82604

**Published:** 2025-04-19

**Authors:** Mary Claire Casper, Varun Soti

**Affiliations:** 1 Obstetrics and Gynecology, Lake Erie College of Osteopathic Medicine, Elmira, USA; 2 Pharmacology and Therapeutics, Lake Erie College of Osteopathic Medicine, Elmira, USA

**Keywords:** ectopic pregnancy, fertility preservation, intrauterine pregnancy, recurrent ectopic pregnancy, salpingotomy

## Abstract

Ectopic pregnancy poses significant risks to future fertility and recurrence, and can be surgically resolved if the traditional approach is ineffective. Salpingotomy has emerged as a preferred surgical option to treat ectopic pregnancy, as it has been shown to preserve fertility and prevent recurrence. This meta-analysis assessed whether salpingotomy impacts the intrauterine pregnancy (IUP) and recurrent ectopic pregnancy (REP) rates. A literature search was conducted using PubMed, MEDLINE (Medical Literature Analysis and Retrieval System Online), Ovid Discovery, and ClinicalTrials.gov. The studies meeting the inclusion criteria were reviewed, and data from 2,220 patients were pooled. The statistical analysis was carried out using the Comprehensive Meta-Analysis Software version 4 (Biostat, Inc., Englewood, New Jersey, United States). The results showed that following salpingotomy, patients reported significantly higher IUP rates (95%CI: 0.487-0.724, p=0.000). The data analysis indicated significant variation in REP rates across the studies, suggesting a high probability of patients not experiencing REP (mean effect size=0.109, 95%CI: 0.074-0.157, p=0.03). A sub-analysis of factors was also conducted, including the impact of age, follow-up time, year of study publication, and geographic location on the IUP and REP rates following salpingotomy. There was a significantly higher number of IUPs in studies published before 2020 than those published after 2020 (mean effect size=0.598, 95%CI: 0.495-0.694, p=0.013). Also notable was a significantly higher IUP rate in patients under 30 (mean effect size=0.58, 95% CI: 0.442-0.706, p=0.007). There was no significant difference in IUP rates due to follow-up time or geographic location (mean effect size=0.613, 95%CI: 0.482-0.730, p=0.964; mean effect size=0.612, 95%CI: 0.541-0.681, p=0.341). Furthermore, REP rates were significantly higher in studies with a follow-up time longer than three years (mean event rate=0.127, 95%CI: 0.098-0.162, p=0.005). There was no significant difference in REP rates across geographic locations, age, or year of publication (p=0.380, p=0.257, and p=0.134, respectively). Overall, salpingotomy provides a higher likelihood of IUP in patients below the age of 30 and has a low risk of REP. The findings underscore the importance of individualized patient counseling, balancing the benefits of salpingotomy for fertility preservation against the risks of REP.

## Introduction and background

Ectopic pregnancy, defined as the implantation of a fertilized ovum outside the uterine cavity, is a significant cause of maternal morbidity and mortality worldwide [1,2]. Approximately 1-2% of all pregnancies are ectopic and implant in the fallopian tube (95%) [3]. Other locations include the ovary, abdominal cavity, cervix, liver, rectum, and pelvic wall [4]. Risk factors include prior ectopic pregnancy, pelvic inflammatory disease, tubal surgery, assisted reproductive technologies, and intrauterine device use. Smoking, advanced maternal age, and a history of infertility also contribute to increased risk [5]. 

Across the globe, ectopic pregnancy remains a prevalent obstetric complication. Once implanted, the trophoblast invades the ectopic site, resulting in vascular damage and potential rupture [6]. Patients with ectopic pregnancy usually show symptoms between six and 10 weeks of gestation. Common manifestations include severe abdominal pain, amenorrhea, and vaginal bleeding. This condition can have a significant impact on patients’ health and requires prompt medical attention [7].

Early detection of ectopic pregnancy is crucial. Monitoring beta-human chorionic gonadotropin (β-hCG) levels is helpful, but it is essential to remember that transvaginal ultrasound is the gold standard for accurate diagnosis [8,9]. Early detection enables expectant or medical management, such as a methotrexate-based regimen, which can provide a more positive outlook for patients. In contrast, a late diagnosis or lack of treatment may lead to severe complications, including rupture of the fallopian tube, massive hemorrhage, and even death in 5-10% of cases. However, positive outcomes with early and effective management are possible [7,10]. 

According to the Centers for Disease Control and Prevention, complications of ectopic pregnancy account for approximately 3.8% of maternal deaths in the United States, making it the leading cause of maternal death in the first trimester. Reducing mortality rates requires the timely administration of treatment [11]. In recent decades, researchers have made significant advancements in treatment methods that now incorporate enhanced surgical techniques, improving patient outcomes for those who have failed expectant/medical management or suffered a rupture [12,13].

The traditional surgical approach for the management of ectopic pregnancy is salpingectomy. It involves the complete removal of the fallopian tube. Surgeons also utilize this procedure to address various other conditions, such as tubal infections (like hydrosalpinx or pyosalpinx), tubo-ovarian abscesses, tubal or ovarian malignancy, and reducing cancer risk in individuals with breast cancer gene mutations. They may perform salpingectomy unilaterally or bilaterally, often using laparoscopic techniques due to their minimally invasive nature; however, they may opt for open laparotomy in urgent or complex cases. Potential complications include injury to surrounding organs, bleeding, infection, and the formation of adhesions. While individuals can preserve fertility with unilateral salpingectomy, they will become sterile after bilateral salpingectomy unless they use assisted reproductive technologies [14].

Surgeons have increasingly favored salpingotomy. This procedure involves making an incision in the fallopian tube to remove an ectopic pregnancy while preserving the tube. More specifically, surgeons make a linear incision over the ectopic site on the antimesenteric border of the fallopian tube to extract the ectopic tissue and then allow the incision to heal by secondary intention or suture it closed. They achieve hemostasis using bipolar cautery or other energy-sealing devices and irrigate the tube to remove residual trophoblastic tissue. A thorough inspection of the abdominal cavity is then conducted to check for bleeding or retained tissue. After the procedure, medical professionals monitor serum β-hCG levels to confirm the complete resolution of the ectopic pregnancy, as persistent trophoblastic tissue may require further intervention, such as methotrexate or additional surgery [14]. Although salpingotomy carries an increased risk of recurrent ectopic pregnancy (REP), it is particularly advantageous for maintaining fertility, an important consideration for many patients [15].

In recent years, researchers have investigated long-term fertility outcomes and REP after salpingotomy. The fertility outcomes seem to vary greatly, with some studies demonstrating pregnancy rates as high as 62.3%, while others show rates as low as 36% after salpingotomy. Similarly, the rate of REP varies widely across studies, ranging from 2.8% to 18.3%. This significant gap in outcomes underscores the need for further research to gain a deeper understanding of the effects of salpingotomy [16].

This meta-analysis assessed and analyzed the current literature on salpingotomy, with a focus on intrauterine pregnancy (IUP) and REP rates. Additionally, it examined how factors such as age, year of publication, geographic location of the study, and total follow-up time of participants influenced these outcome measures. By shedding light on these factors, this review examined the impact of salpingotomy on patients’ fertility and care. The findings of this meta-analysis can guide clinicians in making informed decisions about the treatment of ectopic pregnancy, helping them weigh the potential benefits of preserving fertility against the increased risk of REP. This review aimed to provide valuable data on treating ectopic pregnancy and enhancing outcomes tailored to patients’ specific needs.

## Review

Methods 

We followed the Preferred Reporting Items for Systematic Reviews and Meta-Analyses (PRISMA) guidelines [17] and utilized the Newcastle-Ottawa quality assessment [18].

Search Strategy

We searched the literature using digital databases, including PubMed, MEDLINE (Medical Literature Analysis and Retrieval System Online), ClinicalTrials.gov, and Ovid Discovery. Our search strategy included the terms (“ectopic pregnancy” OR “tubal pregnancy”) AND (“salpingotomy”) AND (“fertility”) AND (“recurrent ectopic pregnancy”). To enhance our literature review, we also examined the reference lists of the selected articles for the meta-analysis. To ensure comprehensive analysis, we did not restrict the search by language, country, or publication date, and assigned the level of clinical evidence to the selected studies [19].

Study Selection

The inclusion criteria included prospective or retrospective studies that reported the IUP, REP, or both, that included women who tried to conceive naturally without using in vitro fertilization, and were peer-reviewed. The exclusion criteria were as follows: (i) studies that included women who underwent in vitro fertilization in their total pregnancy count, (ii) studies that did not assess IUP or REP, and (iii) in vivo studies, ex vivo studies, meta-analyses, systematic reviews, and case reports. 

Data Collection

Two reviewers selected the final included studies using the inclusion and exclusion criteria described above. They gathered general information about the included studies, such as country, trial length, sample size, and study design. The two key outcome measures collected from each study were the IUP rate by natural conception and the REP rate. Additional demographic information, such as age, was collected for subgroup analysis. 

Statistical Analysis

Statistical analyses were performed using Comprehensive Meta-Analysis Software version 4 (Biostat, Inc., Englewood, New Jersey, United States). The mean event rate and 95% confidence interval (CI) with an alpha level (α) of 0.05 were calculated to evaluate the study outcomes. The degree of heterogeneity (I²) was assessed using the test. An I² > 50% and a probability value (p) < 0.1 indicated significant heterogeneity, prompting the use of a random-effects model. In contrast, I² < 50% and p > 0.1 suggested low heterogeneity, which led to the application of a fixed-effects model. Sensitivity analysis was conducted by removing one study at a time to address the heterogeneity in results and pooling the mean effect size. Subgroup analysis was also performed to identify the causes of heterogeneity in the study. Analyses with a p-value < 0.05 were considered statistically significant.

**Figure 1:**
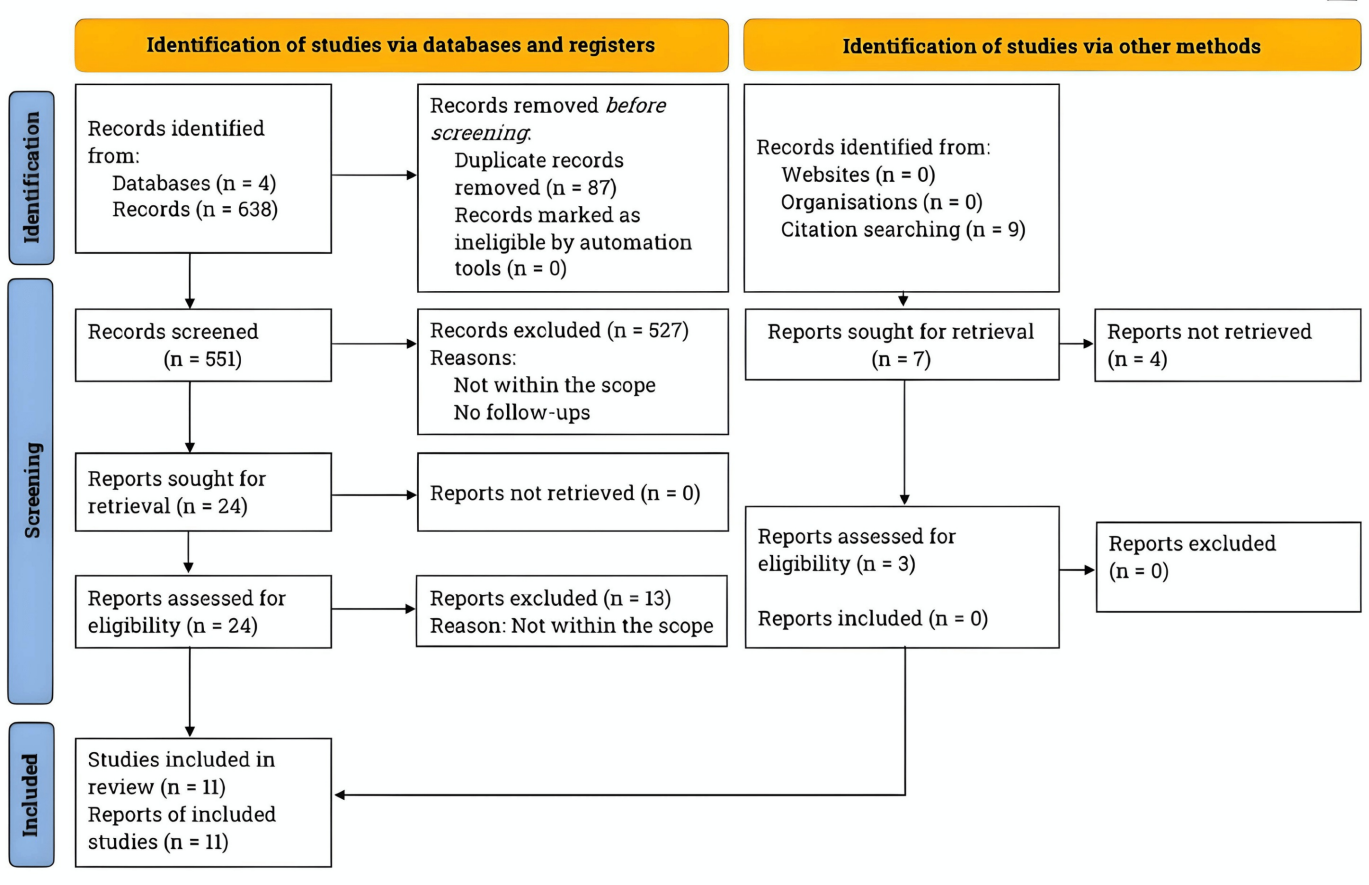
PRISMA flowchart for literature search and study selection PRISMA: Preferred Reporting Items for Systematic Reviews and Meta-Analyses Fotor (California, United States), an online AI application, was utilized to enhance the image’s resolution

The PRISMA flowchart illustrating the process of literature search and study selection is shown in Figure 1.

Results 

The literature search identified 551 articles after removing 81 duplicate entries. Of these, 539 were excluded based on abstract screening. Ultimately, 24 studies met the criteria; however, only 11 fully met the necessary inclusion criteria for this analysis. These studies included a total of 2,220 patients who underwent salpingotomy. Among this patient cohort, there were 890 reported intrauterine pregnancies and 238 cases of recurrent ectopic pregnancies [20-30]. Table 1 gives a summary of the general characteristics of the studies included in this analysis.

**Table 1:** General characteristics of included studies IUP: intrauterine pregnancy; PCS: prospective cohort study; RCS: retrospective cohort study; REP: recurrent ectopic pregnancy; NA: not available

Author(s)	Year	Study design	Country	Sample size	Outcome (IUP)	Outcome (REP)	Average age (years)	Average follow-up time (months)
Asgari et al. [20]	2021	RCS	Iran	52	16	4	30.70	24
Poordast et al. [21]	2022	RCS	Iran	95	35	16	26.49	12-84
Kostrzewa et al. [22]	2013	RCS	Poland	22	11	3	28.9	24
Baggio et al. [23]	2021	RCS	Italy	2	1	0	38.13	12-78
Li et al. [24]	2015	RCS	China	112	57	7	28.80	24
Tavoli et al. [25]	2020	RCS	Iran	106	63	3	NA	24
Silva et al. [26]	1993	PCS	United States	60	36	11	28.6	60
Chen et al. [27]	2017	RCS	China	47	30	7	27.17	36
de Bennetot et al. [28]	2012	RCS	France	646	491	NA	NA	24
Dalkalitsis et al. [29]	2006	RCS	Greece	69	57	7	28.4	120
Turan [30]	2011	RCS	Turkey	63	55	NA	NA	24

Table 2 presents the evaluation of the included studies using the Newcastle-Ottawa Scale.

**Table 2:** Newcastle-Ottawa assessment of included studies * represents a score of 1; – represents a score of 0

Author(s)	Representativeness of exposed cohort	Ascertainment of exposure	Outcome of interest (not present at start)	Comparability by study design	Assessment of outcome	Follow-up length	Adequacy of follow-up	Score
Asgari et al. [20]	*	*	*	*	*	*	*	7
Poordast et al. [21]	*	*	*	*	*	*	*	7
Kostrzewa et al. [22]	*	*	*	*	*	*	*	7
Baggio et al. [23]	*	*	*	*	*	*	*	7
Li et al. [24]	*	*	*	*	*	*	*	7
Tavoli et al. [25]	*	*	*	–	*	*	*	6
Silva et al. [26]	*	*	*	–	*	*	*	6
Chen et al. [27]	*	*	*	*	*	*	*	7
de Bennetot et al. [28]	*	*	*	*	*	*	*	7
Dalkalitsis et al. [29]	*	*	*	*	*	*	*	7
Turan [30]	*	*	*	*	*	*	*	7

Analysis of IUP Following Salpingotomy

**Figure 2:**
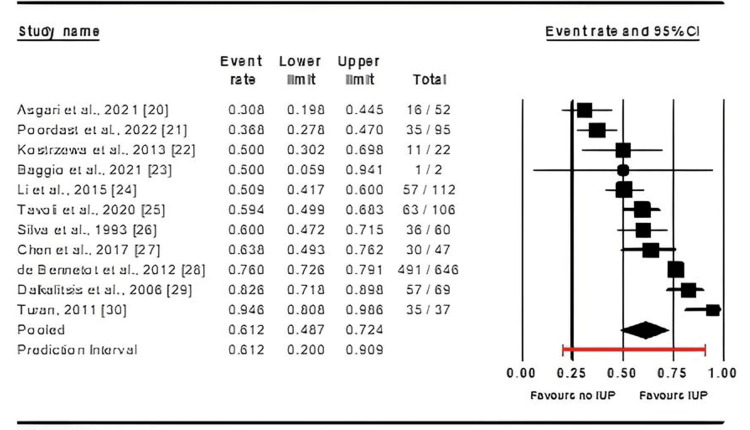
Forest plot illustrating the impact of salpingotomy on intrauterine pregnancy (IUP) rates References: [20-30]

The data analysis revealed that the mean effect size of IUP for patients who underwent salpingotomy was 0.612, indicating that, on average, 61.2% of patients achieved an IUP (95%CI: 0.487-0.724, p = 0.000) (Figure 2).

**Figure 3:**
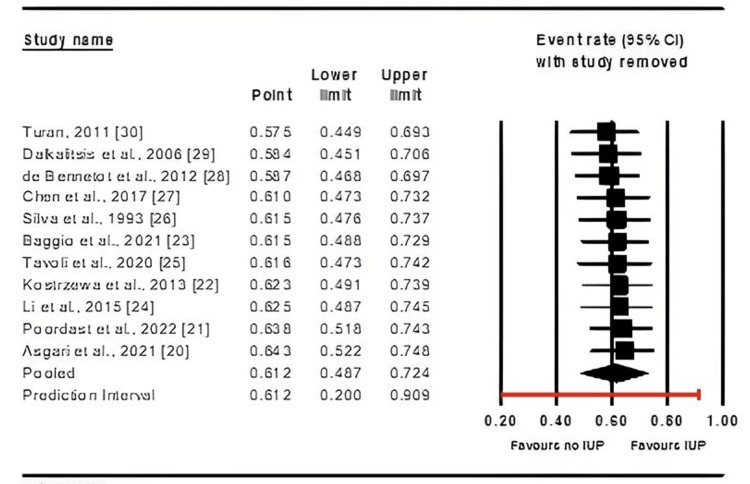
Sensitivity analysis of studies assessing the impact of salpingotomy on intrauterine pregnancy (IUP) rates. References: [20-30]

The observed heterogeneity between studies was substantial, with an I² value of 91.5%, necessitating the use of a random-effects model. The IUP rate following salpingotomy exhibited significant variability among the studies (p < 0.001). A sensitivity analysis was conducted to ensure that these results were not unduly influenced by any single study, yielding a p-value of < 0.001, which reinforced the validity of these findings (Figure 3).

A subgroup analysis was conducted to evaluate whether specific factors, namely age, follow-up duration, publication year, and geographic location of the studies, influenced the IUP rates following salpingotomy.

Age subgroup analysis: The analysis was conducted with six studies categorized in the “average age greater than 30” group [20,23] and two studies in the “average age less than 30” group [21,22,24,26,27,29]. The age cut-off of 30 years was selected because the probability of successful conceptions declines after this age [31]. Given the high heterogeneity (I² = 83.8%, p < 0.001), a random-effects model was employed. The results indicated a higher likelihood of IUP after salpingotomy in women under 30 (mean effect size = 0.58, 95%CI: 0.442-0.706, p = 0.007).

Follow-up time subgroup analysis: This analysis encompassed six studies with “an average follow-up time of less than three years” [22,24-25,28-30] and five studies with “an average follow-up time of more than three years” [21,23,26,27,29]. Due to high heterogeneity (I² = 91.5%, p < 0.001), a random-effects model was utilized. Follow-up duration did not significantly influence IUP rates after salpingotomy (mean effect size = 0.613, 95%CI: 0.482-0.730, p = 0.964).

Year of publication subgroup analysis: Of the studies reviewed, seven were published before 2020 [22,24,26-30], while four studies were published after 2020 [20,21,23,25]. Due to high heterogeneity, the random-effects model was employed again (I² = 91.5%, p < 0.001). The analysis revealed that IUP rates after salpingotomy were significantly higher in studies published before 2020 (the mean effect size = 0.598, 95%CI: 0.495-0.694, p = 0.013).

Geographic location subgroup analysis: In this analysis, two studies originated from Asia [24,27], four from Europe [22,23,28,29], four from the Middle East [20,21,25,30], and one from the United States [26]. High heterogeneity was observed within this subgroup, which warranted the use of a random-effects model (I² = 91.5%, p < 0.001). The results indicated no statistically significant differences in IUP rates after salpingotomy across different geographic locations (mean effect size = 0.612, 95%CI: 0.541-0.681, p = 0.341).

**Figure 4:**
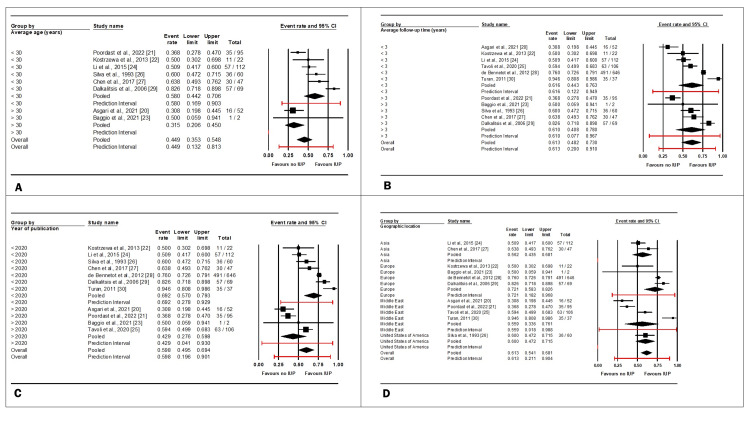
Subgroup analysis of factors including average age (A), follow-up time (B), year of publication (C), and geographic location (D) on intrauterine pregnancy (IUP) rates following salpingotomy References: [20-30]

Figure 4A-4D shows the subgroup analysis, illustrating the impact of these factors on IUP following salpingotomy.

Analysis of REP Following Salpingotomy

**Figure 5:**
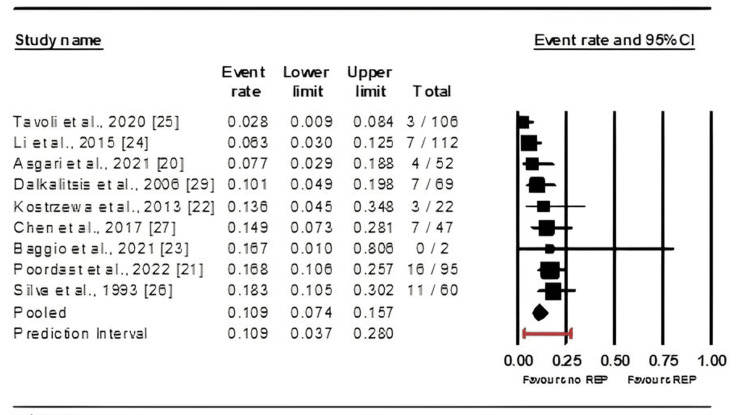
Forest plot demonstrating the impact of salpingotomy on recurrent ectopic pregnancy (REP) rates. References: [20-27,29]

Nine of the 11 studies reviewed reported instances of REP following salpingotomy [20-27,29]. The data analysis revealed a significant variation in REP rates across the studies, indicating a high probability that patients may not experience REP (mean effect size = 0.109, 95%CI: 0.074-0.157, p = 0.039) (Figure 5).

**Figure 6:**
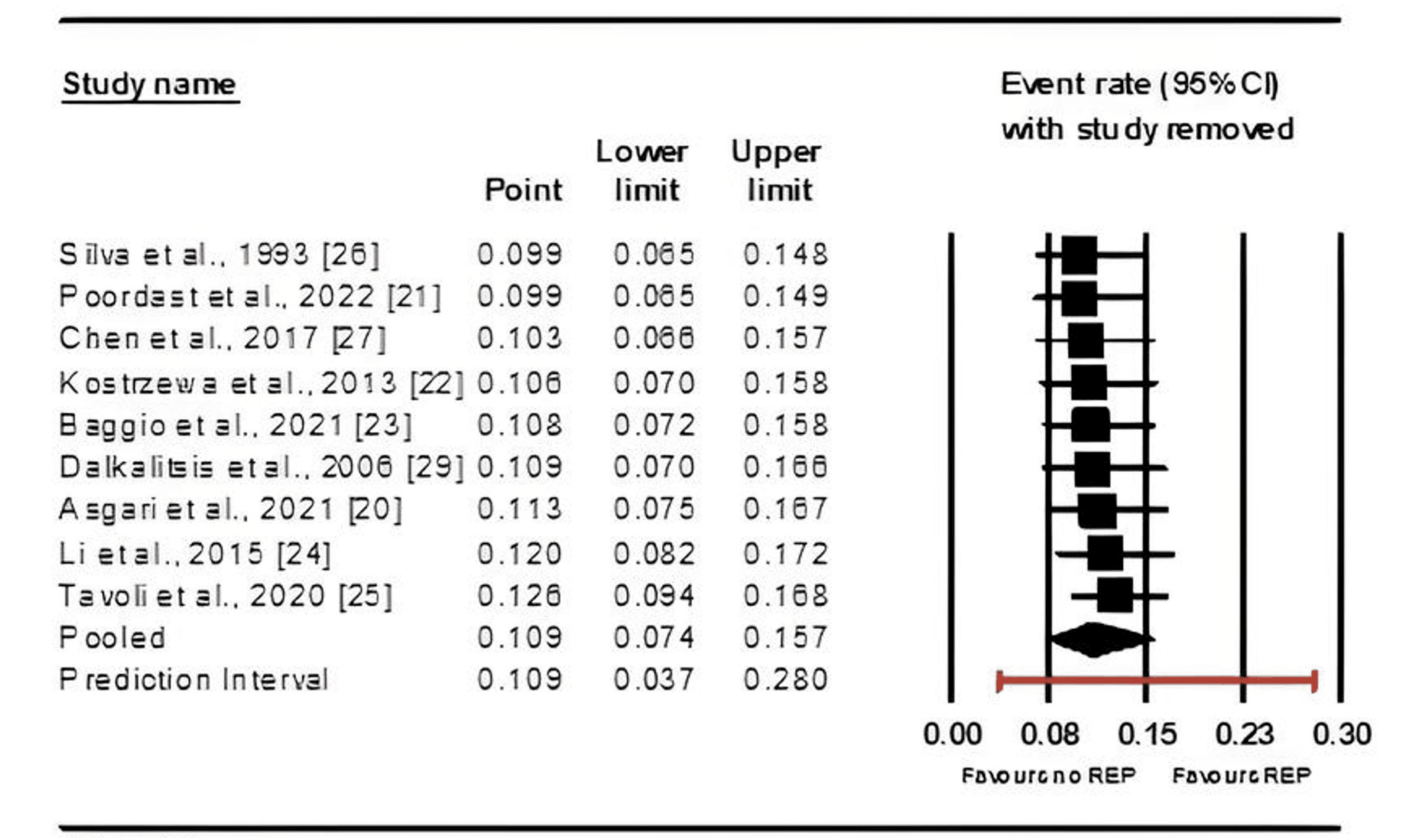
Sensity analysis of studies assessing the impact of salpingotomy on recurrent ectopic pregnancy (REP) rates References: [20-27,29]

Furthermore, a sensitivity analysis corroborated these findings (Figure 6).

A subgroup analysis was conducted to evaluate the impact of age, follow-up time, year of publication, and geographic location on the REP rates following salpingotomy across the studies.

Age subgroup analysis: The group with “an average age of less than 30” included six studies [21,22,24,26,27,29], while two studies were categorized in the group with “an average age of greater than 30” [20,23]. Low heterogeneity was observed (I² = 21.7%, p = 0.257); therefore, a fixed-effects model was used. The analysis revealed no significant difference in REP rates between the two age groups, with a mean event rate of 0.129 (95%CI: 0.090-0.167, p = 0.257).

Follow-up time subgroup analysis: Four studies were included in the “average follow-up time of less than three years” group [20,22,24,25], while five studies comprised the “average follow-up time of more than three years” group [21,23,26,27,29]. Given the moderate heterogeneity, a random-effects model was utilized (I² = 50.7%, p = 0.039). A statistically significant difference in REP rates was observed between the two groups, with the “average follow-up time of more than three years” group exhibiting an increased number of REPs (the mean event rate=0.127, 95%CI: 0.098-0.162, p=0.005).

Year of publication subgroup analysis: Among the nine studies included in this analysis, four were in the “published before 2020” group [22,26,27,29], and five were in the “published after 2020” group [20,21,23-25]. With moderate heterogeneity detected, a random-effects model was applied (I² = 50.7%, p = 0.039). No statistically significant difference in REP rates was found between the two groups (p = 0.134).

Geographic location subgroup analysis: The studies were distributed geographically, with two from Asia [24,27], three from Europe [22,23,29], three from the Middle East [20,21,29], and one from the United States [26]. Given the moderate heterogeneity, a random-effects model was employed (I² = 50.7%, p = 0.039). The analysis revealed no significant difference in REP rates following salpingotomy across the different geographic regions (p = 0.380).

**Figure 7:**
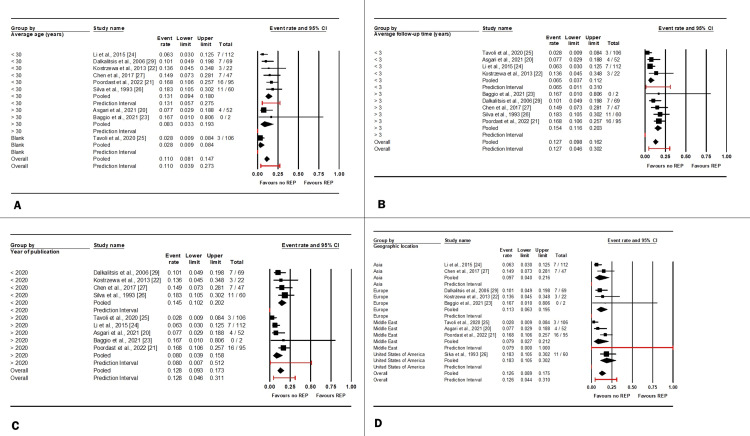
Subgroup analysis of factors including average age (A), follow-up time (B), year of publication (C), and geographic location (D) on recurrent ectopic pregnancy (REP) rates following salpingotomy References: [20-27,29]

Figures 7A-7D present the subgroup analysis, demonstrating the impact of these factors on the REP following salpingotomy.

**Figure 8:**
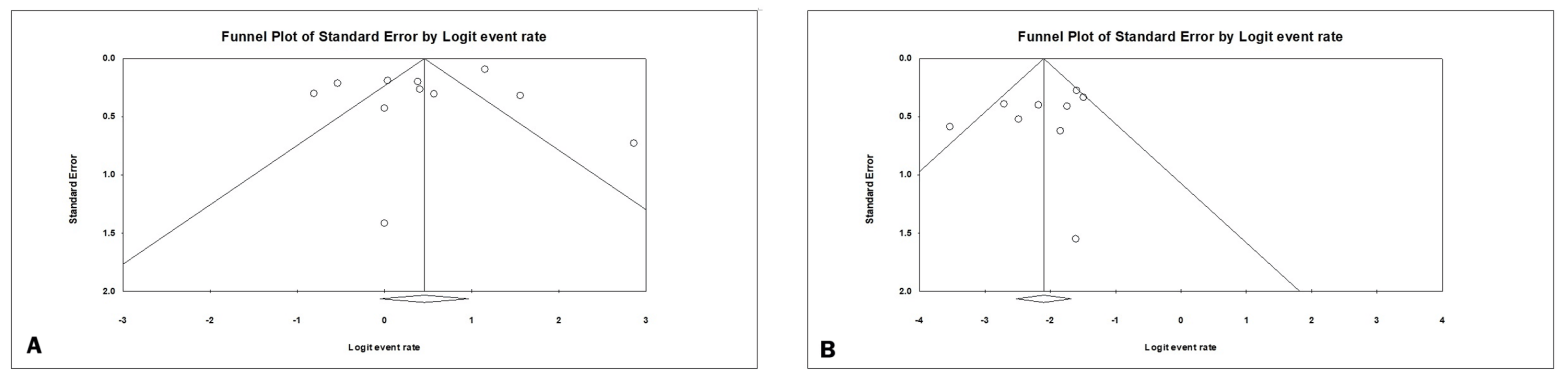
Funnel plots demonstrating publication bias concerning the impact of salpingotomy on intrauterine rates (A) and recurrent pregrnancy rates (B).

Figures 8A-8B illustrate the evaluation of publication bias.

Discussion

This meta-analysis provides a comprehensive evaluation of fertility outcomes following salpingotomy, with a particular focus on IUP and REP rates. The findings offer valuable insights into the potential benefits and risks associated with this fertility-preserving surgical approach.

Fertility Outcomes After Salpingotomy

The pooled IUP rate after salpingotomy was 61.2%, indicating that the majority of patients achieved a successful IUP. However, significant heterogeneity was observed among the included studies, suggesting that various factors may influence the IUP rate. Subgroup analyses were conducted to investigate the role of patient age, study follow-up duration, year of publication, and geographic location [21-30].

Age and IUP rates: The subgroup analysis revealed that women under the age of 30 had a higher likelihood of achieving IUP compared to those over 30, with an average rate of 58%. This is encouraging for younger women seeking to maintain their fertility. However, when compared to the general population, where fertility in women under 35 was approximately 85% over 12 months, there was a notable decline in fertility of about 27% after salpingotomy [20-24,26,27,29]. This suggests that while the procedure preserves fertility, it does not fully restore reproductive potential. Additionally, women over the age of 30 had only a 12.4% chance of achieving an IUP, further emphasizing the need for careful consideration of fertility preservation options in this group [16].

Follow-up duration and IUP rates: Interestingly, the length of follow-up did not significantly influence the IUP rates, suggesting that the majority of successful pregnancies occur within a relatively short time frame following salpingotomy [21-30]. This finding contrasts with previous research, which suggested that IUP rates tend to be higher after salpingotomy with a follow-up period exceeding three years [32]. This is a critical consideration for clinicians counseling patients about their reproductive timeline and expectations.

Publication year and IUP rates: Studies published before 2020 reported significantly higher IUP rates compared to those published more recently [20-30]. While advancements in technology and surgical techniques might suggest improved outcomes, the global decline in fertility due to socioeconomic factors could explain this unexpected trend [33]. A decreasing number of women attempting pregnancy could contribute to lower reported IUP rates in recent studies, indicating that external demographic factors must be considered when interpreting these findings.

Geographic location and IUP rates: No statistically significant difference was observed in IUP rates based on the geographic location of the study [20-30]. This suggests that the benefits of salpingotomy in preserving fertility are broadly applicable across diverse populations and healthcare settings.

Risk of REP After Salpingotomy

While salpingotomy preserves the fallopian tube, it carries the inherent risk of REP, which was found to occur in approximately 10.9% of cases; however, this difference was not statistically significant. Similar to IUP rates, REP rates varied considerably among studies, necessitating subgroup analyses to determine potential influencing factors [20-27,29].

Age and REP rates: Unlike IUP rates, age did not significantly affect REP rates. This indicates that once an ectopic pregnancy has occurred, the risk of recurrence after salpingotomy is relatively independent of maternal age [20-27,29]. Nevertheless, clinicians must carefully monitor these patients regardless of their reproductive age.

Follow-up duration and REP rates: A significant difference in REP rates was observed based on follow-up duration, with more extended follow-up periods (>3 years) associated with a higher incidence of REP [20-27,29]. This highlights the potential underestimation of REP rates in studies with shorter follow-up periods. Previous research has suggested that follow-up duration does not significantly impact REP rates, which conflicts with our findings [32]. More extensive and standardized long-term studies are needed to clarify this discrepancy and determine the actual recurrence risk over time.

Publication year and REP rates: The publication year did not significantly influence REP rates, suggesting that advancements in surgical technique or postoperative management have not markedly altered the likelihood of recurrence over time [20-27,29]. Further research is necessary to determine whether recent surgical modifications or additional treatments can help mitigate the risk of REP.

Geographic location and REP rates: No significant differences in REP rates were found across different geographic regions, indicating that the risk of recurrence is consistent worldwide [20-27,29]. This supports the generalizability of our findings and suggests that factors intrinsic to the surgical procedure and patient characteristics might be more critical determinants of REP risk than regional variations in healthcare delivery.

Clinical implications 

The findings of this meta-analysis have important clinical implications for the management of ectopic pregnancy. The relatively high IUP rate following salpingotomy supports its use as a fertility-preserving option, particularly for younger women who desire future pregnancies. However, the associated risk of REP necessitates a cautious approach, with thorough patient counseling regarding the potential for recurrence and the need for early pregnancy monitoring in subsequent conceptions.

Given that the risk of REP persists over time, patients should be informed about the signs and symptoms of ectopic pregnancy and undergo early ultrasonographic evaluation in future pregnancies. Clinicians should also consider individual patient factors, including age and reproductive goals, when determining the most appropriate management strategy.

Limitations and future directions 

This meta-analysis presents some limitations that warrant consideration. Firstly, the included studies exhibited substantial heterogeneity. Secondly, variations in study design, patient populations, and follow-up durations may have negatively impacted the results. Thirdly, critical factors such as tubal patency, surgical technique, and postoperative management were not consistently reported across the studies, which hindered a thorough analysis of their influence on fertility outcomes. Furthermore, a notable limitation of this analysis is the exclusive inclusion of cohort studies. Given the scarcity of current research on this topic, few randomized controlled trials (RCTs) specifically address outcomes related to IUP and REP following salpingotomy. The absence of RCTs introduces additional bias, as study participants were not randomly assigned to groups. It is plausible that the cohorts within these studies did not fully represent the general population.

Future research should prioritize standardizing the key variables to facilitate more robust study comparisons. Additionally, prospective studies that evaluate the efficacy of adjunctive treatments, such as methotrexate or postoperative hormonal therapy, in mitigating the risk of REP may yield valuable insights into optimizing outcomes after salpingotomy.

## Conclusions

This meta-analysis provides strong evidence that salpingotomy is an effective fertility-preserving surgical option for patients with tubal ectopic pregnancy, with a pooled IUP rate of 61.2% following salpingotomy. While age significantly influenced IUP rates, it did not impact REP rates. Longer follow-up duration was associated with a higher likelihood of recurrence, emphasizing the need for ongoing patient education and monitoring. The findings underscore the importance of individualized patient counseling, balancing the benefits of fertility preservation against the risks of REP.
